# Environmental Risk Factors in Han and Uyghur Children with Dyslexia: A Comparative Study

**DOI:** 10.1371/journal.pone.0159042

**Published:** 2016-07-14

**Authors:** Hua Zhao, Baoping Zhang, Yun Chen, Xiang Zhou, Pengxiang Zuo

**Affiliations:** 1 Medical School, University of Shihezi, Xinjiang, China; 2 Medical School, Quzhou College of Technology, Zhejiang, China; University of Perugia, ITALY

## Abstract

**Background:**

Several studies have been conducted to explore risk factors for dyslexia. However, most studies examining dyslexia have been skewed toward Western countries, and few have considered two nationalities simultaneously. This study focused on differences in dyslexia prevalence and potential environmental risk factors between Han and Uyghur children.

**Methods:**

A cross-sectional study was conducted in Kashgar and Aksu, cities in Xinjiang province, China. A two-stage sampling strategy was used to recruit 2,854 students in grades 3–6 from 5 primary schools in 5 districts; 2,348 valid student questionnaires were included in the analysis. Dyslexia checklists for Chinese and Uyghur children and pupil rating scales were used to identify children with dyslexia. Questions related to the home literacy environment and reading ability were used to evaluate potential environmental risk factors. Single factor analysis and multivariate logistic regression were used to examine prevalence and risk factors for dyslexia.

**Results:**

Dyslexia prevalence differed significantly between Han (3.9%) and Uyghur (7.0%) children (*P* < 0.05), and the boy-to-girl diagnosis ratio was almost 2:1. Multiple logistic regression analysis showed that ethnic differences in dyslexia prevalence between Han and Uyghur children could have occurred because of factors such as mother’s occupation (*P* = 0.02, *OR* = 0.04, 95% *CI* = 0.01–0.68) and the frequency with which parents told stories (*P* = 0.00, *OR* = 4.50, 95% *CI* = 1.67–12.11).

**Conclusions:**

The prevalence of dyslexia was high in all children, particularly those in the Uyghur group. Environmental factors could have been responsible for some of the differences observed. The results contribute to the early identification and management of dyslexia in children from these two groups and research examining developmental dyslexia and differences in racial genetics.

## Introduction

Developmental dyslexia (*DD*), or reading disability, is characterized by impaired reading performance despite adequate motivational, educational, and intellectual opportunities and the absence of sensory or neurological disability[1]. The disorder affects recognition speed, language processing, and understanding. In Western countries, the prevalence of *DD* is estimated at 5–12% in school-age children whose native language is English[2,3]. In China, research into *DD* began relatively late and focused mainly on Han children, for whom the incidence of *DD* was 3.9–8%[4,5]. *DD* is an important and controversial concern worldwide. Research has shown that *DD* initially manifests in the early years of childhood. Although its etiology and pathology remain elusive, there is abundant evidence suggesting that both genetic and environmental factors contribute to *DD*. Genetic association studies have identified several candidate genes associated with *DD*, including *DCDC2*, *DYX1C1*, *ROBO1*, and *KIAA0319*[1,2,6]. These genes can change the structure of protein, which may lead to changes in its functional properties. However, studies in different languages have yielded conflicting results[6–10].

Moreover, cross-sectional studies have shown that some risk factors, such as maternal educational levels, home literacy environment (*HLE*), and socioeconomic status (*SES*), were associated with the treatment of children with *DD* [4,11,12]. However, the results of several other studies have indicated that *SES* factors play a minimal role in *DD* [5]. Moreover, risk factors associated with *DD* could differ across language, race, and culture, resulting in differences between children from different ethnic backgrounds. Xinjiang lies in the far northwest of China, where Central and Western Asian cultures converge. The region is populated by various ethnic groups and predominantly by Uyghur and Han. Because of differences in the nature of the languages spoken and the specificity of the cultures, *DD* prevalences and risk factors differ between Han and Uyghur groups.

With respect to the nature of the languages spoken, Uyghur children are typically educated in both Uyghur and Chinese. The home language (Uyghur) is used until children enter school, but once they reach the third grade, there is a gradual change, whereby Chinese is used in Chinese lessons and Uyghur in others. In contrast, Chinese children are educated only in Chinese. Uyghur belongs to the Turkic language group, which is a branch of the Altaic language family, and uses a phonetic writing system. It involves a completely transparent script and has predictable, bidirectional mapping between orthography and phonology, while Chinese has an ideographic writing system, whereby symbols are used to express words or morphemes in the language, with low transparent orthography [13]. Therefore, children could experience difficulty in one or both languages. To date, knowledge of *DD* in Uyghur children has been limited relative to that of *DD* in children acquiring English (low transparent orthography with inflectional morphology). Results of studies focusing on *DD* in the Han Chinese language (low transparent orthography with isolation or noninflectional morphology) cannot be generalized to Uyghur (high transparent orthography with agglutination in morphology).

In addition, cultural differences could lead to greater diversity in genetic makeup and lower *SES* in Uyghur people relative to that of the Han population. After the People’s Republic of China was founded, many Han people migrated to Xinjiang for military reclamation purposes. For decades, Han distribution was largely in northern Xinjiang, in the Tianshan veins, whereas Uyghur people gathered in the southern area of the province [14]. In addition, Uyghur people follow the Muslim religion, which prohibits marriage to non-Muslims [14], and formed an isolated genetic group. From a wider perspective, Xinjiang remains relatively undeveloped economically, socially, and educationally, and this is particularly evident in the Uyghur group. Uyghur people typically have low household incomes, and the implementation of a more liberal childbirth policy for this group would exacerbate this situation. The latest statistics indicate that there are approximately 1.9 million students in the 3,533 primary schools in Xinjiang province. Although less well researched, *DD* also exists in school-age children of Uyghur ethnicity. Zuo et al. [15] noted that the prevalence of *DD* in Uyghur children was approximately 6.8%. In addition, previous research [4,5,15] has indicated that *DD* prevalence differs between the Han and Uyghur groups. We conducted this study to examine differences in *DD* prevalence and potential environmental risk factors between Han and Uyghur children. We also explored racial differences in *DD* in an attempt to obtain a more accurate pathogenesis of the disorder and provide evidence for further intervention.

## Methods

### Participants

Our study was conducted in the Kashgar and Aksu administrative offices located in the south of Xinjiang province, China, which has a population of 6,685,800 and spans 264,723 square kilometers. In 2013, there were approximately 633,142 students in the 1,552 primary schools in the area. Based on population and economic status, these two regions are classed as medium-sized cities [16]. Therefore, the results of the current study could be generalized to other medium-sized cities in Xinjiang province. As there are numerous primary schools in each region, we used cluster sampling to select 42 primary schools, with a total of 7,452 students in grades 3–6. Of these, we chose 5 Uyghur-Chinese bilingual primary schools, with a total of 2,854 students in grades 3–6, for inclusion in the study.

### Materials

In our cross-sectional survey, the questionnaire included the following: 1) *The Pupil Rating Scale Revised Screening for Learning Disability (PRS)* [17] is a convenient instrument that has been widely used to identify reading disability in China. The questionnaire contains 24 items divided into 5 categories including listening comprehension and memory, language, time and location judgment, athletic ability, and social behavior. It was completed by the head teacher and based on students’ performance at school. The mean score and standard deviation of *PRS* in the present study were 76.77 and 10.21 respectively. 2) *The Dyslexia Checklist for Chinese Children (DCCC)* [18] is a Chinese rating scale for *DD*, with well-established reliability and validity. The questionnaire contains 57 items divided into 8 factors: oral ability, written expression, reading habits, attention, visual perception, writing, hearing, and comprehension. The scale uses 5-point scoring criteria, with 1 indicating that the behavior is never observed, and 5 indicating that it always occurs. The participants’ Chinese parents (either one or both) completed the questionnaire based on the children’s daily performance. Higher scores indicated that the child’s ability was considered poorer. Referring to our norm of *DCCC*, the mean score and standard deviation were 118.87 and 39.75 respectively. 3) *The Dyslexia Checklist for Uyghur Children (DCUC)* was based on the *DCCC* and developed using forward and back translation. The questionnaire was found to have good reliability (retest correlation coefficients from 0.67% to 0.76%) and fair validity (criterion validity correlation coefficients from −0.39 to −0.54). The participants’ Uyghur parents (either one or both) completed the questionnaire based on the children’s daily performance. Referring to our norm of *DCUC*, the mean score and standard deviation were 130.39 and 42.66 respectively. 4) *The Home Literacy Environment and Reading Ability Survey Scale (HLE-RA)* [4] contains three factors: general information, home literacy environment, and children’s reading habits. The participants’ guardians completed the questionnaire to evaluate the children's family background and reading environment. 5) *The China-Wechsler Intelligence Scale for Children (C-WISC)* [19] classifies children into three groups: those with low intelligence (intelligence quotient *[IQ] of* <70), critical state (*IQ* from 70 to 80), and normal intelligence (*IQ of* >80). The mean score and standard deviation of *C-WISC* in the present study were 94.83 and 11.47 respectively.

### Procedure

This research was conducted over two months, November and December 2014, by researchers who were familiar with the scales and experienced in epidemiology. The head teachers gathered all of the parents (either one or both) in classrooms and explained the aim of the study and demands of the questionnaire. The *PRS* scale was completed by the head teachers, while the *DCCC* (for Han children), *DCUC* (for Uyghur children), *HLE-RA* scales were completed by parents (either one or both). Results of the *C-WISC* test indicated that all participants had *IQ* scores in the typical range. Based on the definition of *DD* in the *International Classification of Diseases 10* (*ICD-10*) and *DD* diagnostic criteria in the *Diagnostic and Statistical Manual of Mental Disorders fourth Edition* (*DSM-IV*), we adopted a step-by-step screening method for children with *DD*. The diagnosis of *DD* was based on the following criteria: 1) *PRS* score of <65 points; 2) The score of *DCCC* was two standard deviations higher than the mean scores of Han Chinese children; *DCUC* score was two standard deviations higher than the mean scores of Uyghur children; 3) IQ of >80 assessed via the *C-WISC* test; and 4) no visual and/or auditory disorders or psychiatric diseases.

### Analysis

Following double data entry, the *SPSS12*.*0* package was used to perform the analyses. Descriptive analysis was performed to obtain means ± standard deviations (*Mean ± SD*) for quantitative variables and frequencies for qualitative variables. We used logistic regression analysis to examine the relationship between environmental factors and *DD* diagnosis in different groups. The difference in potential environmental risk factors between Han and Uyghur *DD* children was examined. Variables found to be statistically significant in the univariate analysis were included in a multivariate logistic regression model. All *P* values were two-tailed, with a significance level of 0.05.

### Ethics statement

Prior to initiation of the research, written informed consent was obtained from all participants and their guardians. Moreover, the study was approved by the ethical committee at the Medical Association of Shihezi University.

## Results

### Participant characteristics

Overall, 2,854 students from grades 3–6 in the selected schools agreed to participate in the study. However, only 2,511 students returned the questionnaires, with 2,348 completed appropriately (response rate: 93.5%). The participants’ sociodemographic characteristics are shown in Table 1. Of the 2,348 participants, 1,156 (49.2%) were Han children, 1,192 (50.8%) were Uyghur children, 1,163 (49.5%) were boys, and 1,185 (50.5%) were girls. The children’s average age was 10.9 years for both groups. With respect to family income, 300 (25.2%) Uyghur families and 257 (22.2%) Han families earned incomes of >3000 RMB per month. Similarly, 325 (27.3%) Uyghur families and 352 (30.4%) Han families had incomes of 2000–3000 *RMB*, 343 (28.8%) Uyghur families and 331 (28.6%) Han families earned incomes of 1000–2000 *RMB*, and 224 (18.8%) Uyghur families and 216 (18.7%) Han families earned incomes of <1000 *RMB* per month. With respect to parental occupation, 27.9% of Uyghur fathers were farming, forestry, or fishery workers, and 33.0% of Uyghur mothers were classification of inconvenience. In addition, 23.6% of Han fathers were farming, forestry, or fisheries workers, and a higher proportion of Han parents (36.0% of fathers and 44.2% of mothers) were classified as inconvenience, which meant that they did not work or worked freelancers. Regarding parental educational levels, 54.3% of Uyghur fathers and 61.2% of Han fathers were educated to junior high school level or below, and 26.5% of Uyghur fathers and 15.2% of Han fathers were educated to junior college level or above. Similarly, most mothers were educated to junior high school level or below, while 28.1% and 12.8% of Uyghur and Han mothers, respectively, were educated to junior college level or above (Table 1).

**Table 1:** Descriptive statistics for participants (*N* = 2,348).

Variables	Han	Uyghur
	N = 1156; n (%)	N = 1192; n (%)
**Dependent variable: dyslexia**^#^		
Dyslexia	45 (3.9)	84 (7.0)
No-dyslexia	1111 (96.1)	1108 (93.0)
**Sex**		
Boys	599 (51.8)	564 (47.3)
Girls	557 (48.2)	628 (52.7)
**Grade**		
Three	325 (28.1)	298 (25.0)
Four	338 (29.2)	381 (32.0)
Five	341 (29.5)	457 (38.3)
Six	152 (13.1)	56 (4.7)
Age*(years), X±SD	10.94±1.3	10.99±1.1
**Variables of Socio-Economic Status (SES)**		
**Income of family per month (RBM)**		
>3000 Yuan	257 (22.2)	300 (25.2)
2000–3000 Yuan	352 (30.4)	325(27.3)
1000–2000 Yuan	331 (28.6)	343 (28.8)
<1000 Yuan	216 (18.7)	224 (18.8)
**Occupation of father**		
Professional technical staff	101 (8.7)	138 (11.6)
Institution, government and office staff	58 (5.0)	206 (17.3)
Business staff	120 (10.4)	143 (12.0)
Manual workers	188 (16.3)	102 (8.5)
Farming, forestry, fishery worker	273 (23.6)	332 (27.9)
Classification of inconvenience	416 (36.0)	271 (22.7)
**Occupation of mother**		
Professional technical staff	67 (5.8)	105 (8.8)
Institution, government and office staff	39 (3.4)	175 (14.7)
Business staff	123 (10.6)	114 (9.6)
Manual workers	150 (13.0)	75 (6.3)
Farming, forestry, fishery worker	266 (23.0)	329 (27.6)
Classification of inconvenience	511 (44.2)	394 (33.0)
**Father’s education level**		
Junior high school or below	707 (61.2)	647(54.3)
Senior high school or equivalent	273 (23.6)	229 (19.2)
Junior college or above	176 (15.2)	316 (26.5)
**Mother’s education level**		
Junior high school or below	789 (68.3)	625 (52.4)
Senior high school or equivalent	219 (18.9)	232 (19.5)
Junior college or above	148 (12.8)	335 (28.1)

* was represented as Mean±SD deviation

^#^*χ*^*2*^ = 11.3, *P*<0.01

### Dyslexia prevalence rates in boys and girls of different ethnicities

**Fig 1:**
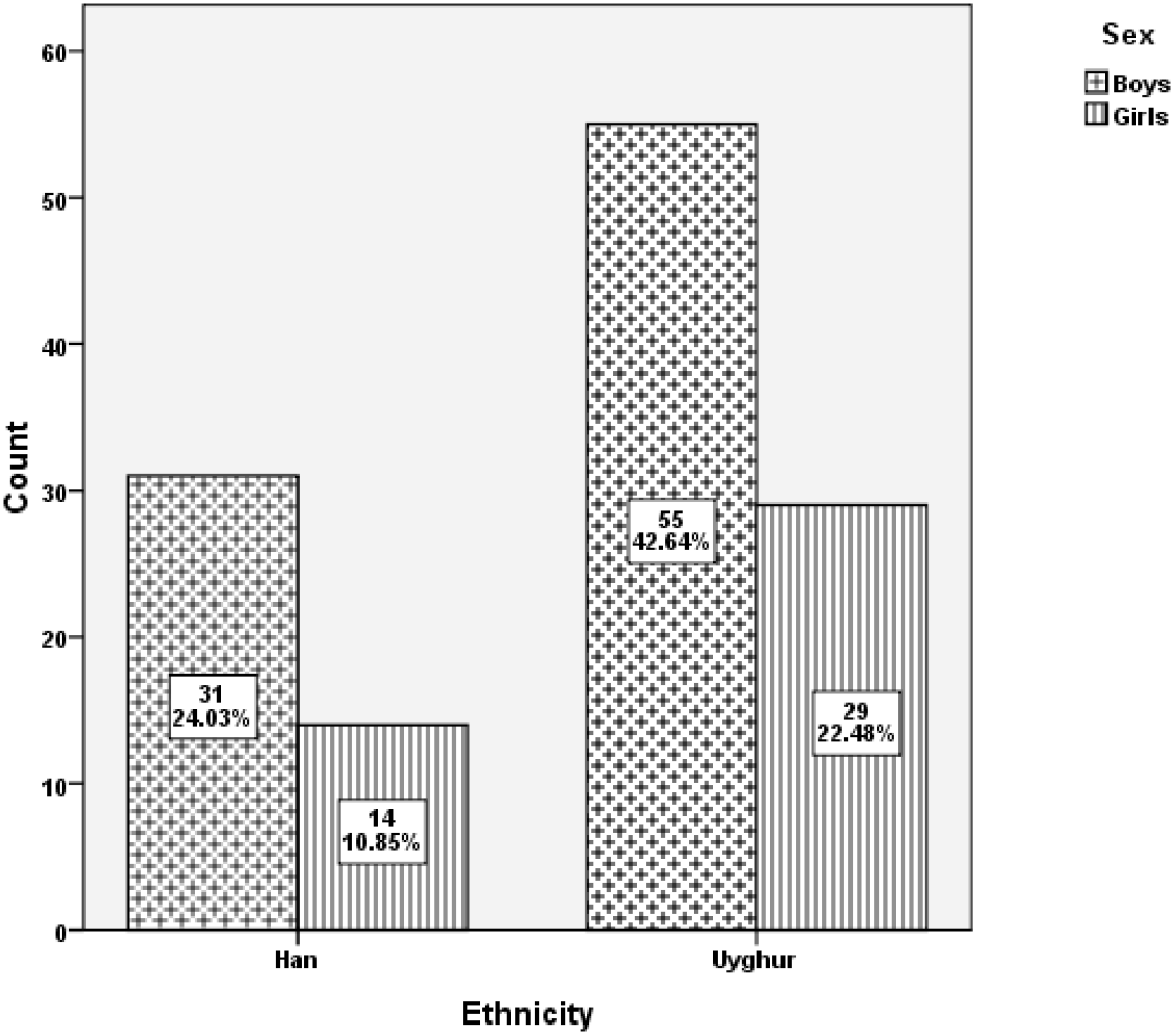
Distribution of dyslexia according to differences ethnicity and sex.

Of 2,348 participants, 129, including 45 Han and 84 Uyghur children, had been diagnosed with *DD*. The *DD* prevalence rates differed significantly between Han (3.9%) and Uyghur (7.0%) children (*χ*^*2*^ = 11.25, *P* < 0.05). The distribution of *DD* according to sex and ethnicity is shown in Fig 1. Of the 129 children with *DD*, 86 were boys and 43 were girls, and the boy-to-girl diagnosis ratio was almost 2:1. The proportions of boys who were Han and Uyghur were 24.03% and 42.64%, respectively. Similarly, 10.85% and 22.48% of the girls were Han and Uyghur, respectively.

### Factors influencing *DD* diagnosis in Han and Uyghur children

We analyzed the potential risk factors for *DD* in Han and Uyghur children in the logistic regression analysis. Factors such as sex, mother’s occupation, parental educational level, and most *HLE* factors differed significantly between children with and without *DD* in both groups (*P* < 0.05). However, this difference was not observed for age in either group. In addition, family income, paternal occupation, and maternal educational level were specifically associated with *DD* in Uyghur children (*P* < 0.05), and child’s educational grade and book-buying expenses were associated with *DD* in Han children (*P* < 0.05; Table 2).

**Table 2:** Analytical statistics for Uyghur and Han children with dyslexia.

Variables	Han Subjects		N = 1156		Uyghur Subjects		N = 1192		All DD Subjects		N = 129	
	DD	No-DD	OR (CI 95%)	P	DD	No-DD	OR (CI 95%)	P	Han-DD	Uyg-DD	OR (CI95%)	P
	n = 45	n = 1111			n = 84	n = 1108			n = 45	n = 84		
	n(%^b^)	n(%^b^)			n(%^b^)	n(%^b^)			n(%^c^)	n(%^c^)		
**Gender**			0.71(0.39–1.23)	0.27			0.45(0.28–0.71)	0.00			0.79(0.37–1.67)	0.54
Boys	31(68.9)	568(51.1)			55(65.5)	509(45.9)			31(5.2)	55(9.8)		
Girls	14(31.1)	543(48.9)			29(34.5)	599(54.1)			14(2.5)	29(4.6)		
**Grade**			0.64(0.46–0.88)	0.01			0.89(0.69–1.14)	0.36			1.50(1.06–2.36)	0.03
Three	18(40.0)	307(27.6)			21(25.0)	277(25.0)			18(5.5)	21(7.1)		
Four	17(37.8)	321(28.9)			31(36.9)	350(31.6)			17(5.0)	31(8.1)		
Five	8(17.8)	333(30.0)			31(36.9)	426(38.4)			8(2.4)	31(6.8)		
Six	2(4.4)	150(13.5)			1(1.2)	55(5.0)			2(1.3)	1(1.8)		
**Age**^a^**(years), X±SD**	10.7±1.2	10.9±1.3	0.89(0.71–1.13)	0.35	10.9±0.9	11.0±1.1	0.95(0.78–1.17)	0.66	10.8±1.2	10.9±0.9	1.18(0.83–1.67)	0.34
**Variables of Socio-Economic Status (SES)**												
**Income of family per month (RBM)**			0.92(0.69–1.23)	0.59			1.31(1.06–1.62)	0.01			1.43(1.18–2.08)	0.04
>3000 Yuan	11(24.4)	246(22.1)			10(11.9)	290(26.2)			11(4.3)	10(3.3)		
2000–3000 Yuan	15(33.3)	337(30.3)			23(27.4)	302(27.3)			15(4.3)	23(7.1)		
1000–2000 Yuan	11(24.4)	320(28.8)			34(40.5)	309(27.9)			11(3.3)	34(9.9)		
<1000 Yuan	8(17.8)	208(18.7)			17(20.2)	207(18.7)			8(3.7)	17(7.6)		
**Occupation of father**			1.07(0.88–1.30)	0.51			1.24(1.08–1.43)	0.00			0.94(0.75–1.18)	0.60
Professional technical staff	4(8.9)	97(8.7)			4(4.8)	134(12.0)			4(4.0)	4(2.9)		
Institution, government and office staff	4(8.9)	54(4.9)			11(13.1)	195(17.6)			4(8.9)	11(15.6)		
Business staff	3(6.7)	117(10.5)			8(9.5)	135(12.2)			3(2.5)	8(5.6)		
Manual workers	5(11.1)	183(16.5)			2(2.4)	100 (9.1)			5(4.8)	2(5.6)		
Farming, forestry, fishery worker	6(13.3)	267(24)			35(41.7)	297(26.8)			6(2.2)	35(10.5)		
Classification of inconvenience	23(51.1)	393(35.4)			24(28.6)	247(22.3)			23(5.5)	24(8.9)		
**Occupation of mother**			1.31(1.02–1.70)	0.03			1.16(1.01–1.33)	0.04			0.71(0.52–0.97)	0.03
Professional technical staff	0(0.0)	67(6.0)			2(2.4)	103(9.3)			0(0.0)	2(1.9)		
Institution, government and office staff	1(2.2)	38(3.4)			8(9.5)	167(15.1)			1(4.4)	8(12.3)		
Business staff	5(11.1)	118(10.6)			2(2.4)	112(10.1)			5(4.1)	2(1.8)		
Manual workers	1(2.2)	149(13.4)			2(2.4)	112(10.1)			1(1.1)	10(26.0)		
Farming, forestry, fishery worker	9(20.0)	257(23.1)			35(41.7)	294(26.5)			9(3.4)	35(10.6)		
Classification of inconvenience	29(64.4)	482(43.4)			27(32.1)	367(33.1)			29(5.7)	27(6.9)		
**Father’s education level**			0.55(0.33–0.93)	0.02			0.65(0.48–0.87)	0.00			1.40(0.81–2.42)	0.22
Junior high school or below	37(82.2)	670(60.3)			59(70.2)	615(54.2)			37(5.2)	59(9.1)		
Senior high school or equivalent	3(6.7)	270(24.3)			10(11.9)	219(19.3)			3(1.1)	10(4.4)		
Junior college or above	5(11.1)	171(15.4)			15(17.9)	301(26.5)			5(5.7)	15(9.2)		
**Mother’s education level**			0.59(0.34–1.02)	0.06			0.59(0.44–0.81)	0.00			1.77(1.16–3.31)	0.02
Junior high school or below	35(77.8)	754(67.9)			59(70.2)	566(51.1)			35(4.4)	59(9.4)		
Senior high school or equivalent	9(20.0)	210(18.9)			12(14.3)	220(19.9)			9(4.1)	12(5.2)		
Junior college or above	1(2.2)	147(13.3)			13(15.5)	322(29.0)			1(1.1)	13(6.5)		
**Home Literacy Environment (HLE)**												
**Frequency of parents telling stories**			0.46(0.26–0.81)	0.01			0.66(0.46–0.95)	0.02			2.54(1.29–5.00)	0.01
Occasionally	33(73.3)	567(51.0)			40(47.6)	365(32.9)			33(5.5)	40(9.9)		
Sometimes	10(22.2)	428(38.5)			39(46.4)	599(54.1)			10(2.3)	39(6.1)		
Often	2(4.4)	116(10.4)			5(6.0)	144(13.0)			2(1.7)	5(3.4)		
**How often parents encourage the child to read extra-curricular books**			0.53(0.36–0.77)	0.00			0.60(0.45–0.81)	0.00			0.94(0.60–1.49)	0.80
Occasionally	17(37.8)	172(15.5)			27(32.1)	206(18.6)			17(9.0)	27(11.6)		
Sometimes	12(26.7)	357(32.1)			35(41.7)	442(39.9)			12(3.3)	35(7.3)		
Often	16(35.6)	582(52.4)			22(26.2)	460(41.5)			16(2.7)	22(4.6)		
**Frequency of parents buying books children are interested in**			0.49(0.33–0.74)	0.00			0.63(0.47–0.84)	0.00			1.52(0.94–2.44)	0.09
Occasionally	22(48.9)	277(24.9)			29(34.5)	218(19.7)			22(7.4)	29(11.7)		
Sometimes	15(33.3)	452(40.7)			31(36.9)	437(39.4)			15(3.2)	31(6.6)		
Often	8(17.8)	382(34.4)			24(28.6)	453(40.9)			8(2.1)	24(5.0)		
**Frequency of parents buying new books**			1.34(1.02–1.91)	0.03			1.13(0.95–1.34)	0.16			0.73(0.62–1.04)	0.08
Per week	1(2.2)	31(2.8)			1(1.2)	73(6.6)			1(3.1)	1(1.4)		
Per month	2(4.4)	97(8.7)			10(11.9)	160(14.4)			2(2.0)	10(5.9)		
Per term	6(13.3)	261(23.5)			11(13.1)	201(18.1)			6(2.3)	11(5.2)		
Every year	0(0.0)	41(3.7)			8(9.5)	38(3.4)			0(0.0)	8(17.4)		
Irregularly	36(80.0)	681(61.3)			54(64.3)	636(57.4)			36(5.0)	54(7.8)		
**Children have regular reading time**			1.89(1.01–3.57)	0.04			1.78(1.14–2.77)	0.01			0.46(0.21–0.97)	0.04
Yes	15(33.3)	541(48.7)			44(52.4)	733(66.2)			15(2.7)	44(5.7)		
No	30(66.7)	570(51.3)			40(47.6)	375(33.8)			30(5.0)	40(9.6)		
**Frequency of parents reading**			1.72(1.06–2.80)	0.02			2.56(1.81–3.65)	0.00			0.84(0.48–1.48)	0.55
Every day	3(6.7)	184(16.6)			9(10.7)	287(25.9)			3(1.6)	9(3.0)		
Every week	8(17.8)	314(28.3)			18(21.4)	438(39.5)			8(2.5)	18(4.0)		
Every month	34(75.6)	613(55.2)			57(67.9)	383(34.6)			34(5.3)	57(13.0)		
**Duration of TV watching for child every day**			1.48(1.11–1.98)	0.01			1.66(1.32–2.09)	0.00			0.94(0.65–1.36)	0.74
Less than 1 hour	10(22.2)	407(36.6)			17(20.2)	414(37.4)			10(2.4)	17(3.9)		
1–2 hour	20(44.4)	464(41.8)			38(45.2)	512(46.2)			20(4.1)	38(6.9)		
2–3 hour	5(11.1)	135(12.2)			18(21.4)	113(10.2)			5(3.6)	18(13.7)		
More than 3 hours	10(22.2)	105(9.5)			11(13.1)	69(6.2)			10(8.7)	11(13.8)		
**Expenses of buying books for child every year**			0.56(0.36–0.90)	0.01			0.87(0.63–1.21)	0.42			0.95(0.59–1.54)	0.84
Less than 150 Yuan	29(64.4)	499(44.9)			60(71.4)	695(62.7)			29(5.5)	60(8.0)		
150–300 Yuan	13(28.9)	441(39.7)			17(20.2)	318(28.7)			13(2.9)	17(5.1)		
300–500 Yuan	2(4.4)	115(10.4)			3(3.6)	68(6.1)			2(1.7)	3(4.2)		
More than 500 Yuan	1(2.2)	56(5.0)			4(4.8)	27(2.4)			1(1.8)	4(12.9)		

^a^ Age was represented as mean± standard deviation

^b^ represented constituent ratio

^c^ represented odds ratio, n/N, means the prevalence of dyslexia under each factors.

We then removed the data for children who had not been diagnosed with *DD*. Table 2 shows the differences in environmental risk factors between Han and Uyghur children with *DD*, based on univariate analysis. The results indicate that the prevalence rates for *DD* were 5.5%, 5.0%, 2.4%, and 1.3% in grades 3, 4, 5, and 6, respectively, in Han children with *DD*; these rates were significantly lower relative to those of Uyghur children with *DD* (*P* < 0.05). There was significant difference in family income between the Han and Uyghur children with *DD* (*P* < 0.05). With respect to income, 4.3%, 4.3%, 3.3%, 3.7% of families of Han children with *DD* earned incomes of >3000 RMB, 2000–3000 RMB, 1000–2000 RMB, and <1000 RMB, respectively. These proportions were lower relative to those of families of Uyghur children with *DD*, of which 3.3%, 7.1%, 9.9%, and 7.6% earned incomes of >3000 RMB, 2000–3000 RMB, 1000–2000 RMB, and <1000 RMB, respectively. Regarding maternal occupation, 26.0% and 1.1% of mothers of Uyghur and Han children with DD, respectively, were service staff members or manual workers. In addition, when mothers were educated to junior college level or above, *DD* prevalence rates differed significantly between the two groups (*P* < 0.05). Moreover, there was significant association between the prevalence difference of *DD* in this two groups and two *HLE* factors (e.g., Frequency of parents telling stories (*P* = 0.01, *OR* = 2.54) and Children have regular reading time (*P* = 0.04, *OR* = 0.46)).

### Multivariate logistic regression analysis of the factors influencing *DD* diagnosis in Uyghur and Han children

Multivariate logistic regression analysis was performed to examine the effect sizes for potential risk factors detected in the univariate analysis (Table 3). For Han children, two risk factors (paternal educational level and frequency with which parents encouraged children to read extracurricular books) were entered into the equation. Similarly, five risk factors (sex; paternal occupation; maternal occupation; frequency with which parents tell stories; and child’s daily TV viewing duration) were entered into the equation for Uyghur children. In addition, we included *DD* prevalence in Uyghur and Han children as a dependent variable and the six potential risk factors as independent variables in the analysis of the sizes of the effects of these factors on *DD* prevalence rates. The results showed that four risk factors (maternal occupation and frequency with which parents tell stories) were significantly associated with *DD* prevalence differences between the Han and Uyghur groups. Relative to the differences in the proportions of Han and Uyghur children with *DD* whose mothers were employed as professional or technical staff, the difference between proportions of Han and Uyghur with *DD* children whose mothers were employed as business staff was greatest (*P* = 0.02, *OR* = 0.04, 95% *CI* = 0.01–0.68). This indicates that in those whose mothers were employed as business staff, Uyghur children were 0.04 times more likely to have *DD* relative to Han children. For parents with occasionally telling stories, the OR to be the prevalence differences of *DD* was 4.5 times as high as those parents only telling story sometimes.

**Table 3:** Multivariate logistic regression analysis of factors associated with dyslexia.

Variables	P	OR	95% CI
**Han Subjects**			
**Father’s education level**			
Junior high school or below		1	
Senior high school or equivalent	0.01	0.23	0.07–0.75
**How often parents encourage the child to read extra-curricular books**			
Occasionally		1	
Sometimes	0.01	0.36	0.17–0.79
Often	0.00	0.32	0.16–0.66
**Uyghur Subjects**			
**Gender**			
Boys		1	
Girls	0.01	0.51	0.31–0.83
**Occupation of father**			
Professional technical staff		1	
Business staff	0.05	3.78	0.96–14.89
Classification of inconvenience	0.04	3.65	1.03–12.92
**Occupation of mother**			
Professional technical staff		1	
Manual workers	0.05	3.79	0.97–14.78
** Frequency of parents reading**			
Every day		1	
Every month	0.00	3.63	1.73–7.65
**Duration of TV watching for child every day**			
Less than 1 hour	0.02	1	
2–3 hour	0.00	2.78	1.34–5.78
More than 3 hours	0.02	2.66	1.14–6.20
**All DD Subjects**			
**Occupation of mother**			
Professional technical staff		1	
Business staff	0.02	0.04	0.01–0.68
**Frequency of parents telling stories**			
Occasionally		1	
Sometimes	0.00	4.50	1.67–12.11

## Discussion

To our knowledge, this study was the first to consider the prevalence of *DD* and associated risk factors in a large sample of Han and Uyghur children. We also explored the environmental risk factors affecting *DD* prevalence differences between the Han and Uyghur groups. Our results showed that *DD* prevalence differed between Han (3.9%) and Uyghur (7.0%) children, and the boy-to-girl diagnosis ratio was almost 2:1. In addition, *SES* and *HLE* factors were significantly associated with *DD* in both groups. Factors such as grade, family income, maternal occupation, maternal educational level, and two *HLE* factors (maternal occupation and frequency with which parents tell stories) could exert an impact on differences in *DD* prevalence between the two groups; this was particularly likely for maternal occupation and frequency with which parents tell stories.

Because of linguistic and genetic differences between Uyghur, Chinese, and other populations, the *DD* prevalence varies between different studies. In European populations, the prevalence of *DD* is 5–12% [2,3]. The corresponding figure for China has been reported as 3.9–8.0% [4]. Zuo et al. showed that the *DD* prevalence in a Uyghur population was estimated at 6.9% [15]. Similarly, our finding that *DD* prevalence in the Han group (3.9%) was significantly lower relative to that of the Uyghur group (7.0%) was consistent with those of previous studies. There are a number of possible reasons for this result. For instance, Uighur children receive bilingual Uyghur and Chinese education, and Uyghur is an alphabetical language with a linear one-dimensional alphabet [20] that differs from Chinese (ideograph language) in linguistic characteristics [21]. In addition, previous studies have shown that the cerebral regions activated by Uyghur and Chinese language are not identical and found that activation in the left anterior cingulate gyrus could be associated with the Uyghur language [20]. Moreover, research focusing on bilingual Chinese and English individuals showed that when children are educated in two languages, the second language is likely to be influenced by the linguistic and cultural conventions of the first and vice versa [22]. In addition, molecular studies have found that the Uyghur population is of typical mixed genetic origin, with 60% and 40% of the population of European and East Asian ancestry, respectively; this may explain, at least in part, the genetic influence on studied *DD* children of Uyghur whose prevalence rate is high than Chinese children but lower than European children [23]. Further, the Uyghur population formed an isolated genetic group, because it was not strongly influenced by recent migration [23], and is overwhelmingly of Muslim faith, which prohibits marriage to non-Muslims [14].

In the present study, although there was no sex difference observed between groups, the boy-to-girl *DD* diagnosis ratio was approximately 1:2 in both Han and Uyghur children, and significant differences were observed between sexes in both groups. In Uyghur children, multivariate logistic regression analysis showed that the proportion of girls with *DD* was 0.51 times that of boys. Our results were consistent with those of Renato, who found that a higher number of boys were diagnosed with *DD* relative to that of girls [24]. However, Liederman and other researchers considered evidence suggesting a preponderance of boys with *DD* controversial and weak and posited that it required support from other studies [25,26]. There are several possible reasons for this difference. Previous analyses have indicated that girls enjoy a small advantage in language production until the age of 36 months [27]. Further, in China, particularly in certain ethnic groups such as the Uyghur, many parents view boys as superior; therefore, education and the way the child is raised will depend largely on the child’s sex. In terms of academic performance, boys are believed to be good at math, and girls are thought to be good at languages and arts. To some extent, this could reflect sex-related differences linked to the location of the speech-processing center and differences in cognitive development. Our research implicating a important change in intervening *DD*, education methods should be selected based on children’s sex because of the sex difference in *DD* diagnosis.

Our research also demonstrated that educational grade exerted a significant influence on *DD* prevalence in Han, but not Uyghur, children. This discrepancy could have occurred because Han children are under greater pressure to enter better middle schools relative to that exerted on Uyghur Children. Moreover, there were significant differences between grades in Han and Uyghur children with *DD*. Further, *DD* prevalence showed a downward trend with progression to higher grades. This could be explained by Shaywitz’s findings indicating that *DD* prevalence was lower in higher grades, relative to that in lower grades, because some *DD* symptoms improve through systematic learning, [28]. However, multiple logistic regression analysis showed that there was no association between grades and risk of *DD* in Han or Uyghur children. Indeed, the association between grade and *DD* prevalence remains unclear. In addition, several studies have shown higher *DD* prevalence in higher grades relative to that observed in lower grades [29]. In order to explore the exact nature of the association between grade and prevalence, further studies should be conducted in the near future.

In previous studies, parental education and occupation and family income represented *SES*, which has been associated with children’s language development [30]. Our results suggest that *SES*-related factors, such as maternal occupation and paternal educational level, were associated with *DD* in Han children. In addition, all *SES*-related factors were associated with *DD* in Uyghur children. Moreover, in both Han and Uyghur groups, the results showed that higher parental educational levels and family income were related to lower risk of *DD*. This result could be explained by previous findings indicating that higher *SES* was related to reduced *DD* prevalence[4]. Further, *DD* prevalence in Han children was significantly lower relative to that observed in Uyghur children in family income and maternal education level factors even though *SES* in Uyghur group are better than that in Han group. This finding could be explained by linguistic and genetic differences between Han and Uyghur populations, which could have led to higher *DD* prevalence in Uyghur children relative to that observed in Han subjects [20–23].

Furthermore, results of multiple logistic regression analysis indicated that ethnic differences in *DD* prevalence between Han and Uyghur children could have occurred because of differences in their mothers’ occupations. Relative to the differences in proportions of Uyghur and Han children whose mothers were employed as professional or technical staff, the difference in the proportions of children whose mothers were employed as business staff was greater. This finding indicates that mothers of Han children with *DD* were employed mainly as business staff, and as such, rarely stayed at home or communicated with their children; therefore, Han children could have received little attention or verbal stimulation, resulting in a developmental lag in reading ability [30]. However, most Uyghur families were involved in farming and animal husbandry, and mothers had more time to take care of their children. Therefore, mothers play an important role in raising children, if mothers remain at home and communicate with their children more frequently, *DD* prevalence is likely to decrease.

*HLE* has been found to be one of the most important modifiable risk factors for *DD* in several studies. Rashid et al. conducted a study involving children with reading disabilities and showed that *HLE* was significantly associated with children’s comprehension and spelling scores [31]. He et al. showed that increasing literacy-related activity and reducing the total time spent using electronic devices was a potentially protective factor for Chinese children at risk of *DD* [11]. Similarly, the results of the present study highlighted this point, as the risk of *DD* decreased in children of both groups if their parents provided a better *HLE*. Further, other factors, such the frequency with which parents tell stories and whether children have regular reading time, were significantly associated with *DD* prevalence in both Han and Uyghur children. In particular, when parents told stories more frequently, the OR for *DD* prevalence differences between groups decreased. However, Rashid et al. showed that parents’, rather than children’s, home literacy activities were significantly associated with several types of academic ability in their children. Therefore, to validate the positive effect of *HLE*-related factors on *DD* in both groups, further studies involving Uyghur populations are required.

This study recruited a large sample from two major ethnic groups in the Kashgar and Aksu regions. We collected socioeconomic and environmental variables that were important in examining *DD*. The results of our study identified risk factors associated with differences in *DD* prevalence between Han and Uyghur children, and statistical significance for some factors linked to the groups. These findings provide important environmental insight into *DD*. However, the study was subject to a number of limitations. For example, there are many risk factors associated with *DD*, and only some of these were analyzed in our study. Details concerning other factors, such as birth history, nursery period, and history of infantile illness, were unknown for the participants in our study. Furthermore, the etiology of *DD* is complex, and related neurology, genetics, and psychology have been investigated in many studies [2,9,10]. The present study considered only environmental risk factors affecting Han and Uyghur children with *DD*. Further research is required to understand the molecular mechanisms underlying *DD* in Uyghur children and whether they differ when compared to other groups.
